# Advances in laparoscopic urologic surgery techniques

**DOI:** 10.12688/f1000research.7660.1

**Published:** 2016-04-21

**Authors:** Haidar M. Abdul-Muhsin, Mitchell R. Humphreys

**Affiliations:** 1 Department of Urology, Mayo Clinic Hospital, Phoenix, Arizona, 85054, USA

**Keywords:** laparoscopic urology, Augmented reality, Laparoendoscopic single site surgery, natural orifice transluminal surgery, Laparoendoscopic, insufflation devices

## Abstract

The last two decades witnessed the inception and exponential implementation of key technological advancements in laparoscopic urology. While some of these technologies thrived and became part of daily practice, others are still hindered by major challenges. This review was conducted through a comprehensive literature search in order to highlight some of the most promising technologies in laparoscopic visualization, augmented reality, and insufflation. Additionally, this review will provide an update regarding the current status of single-site and natural orifice surgery in urology.

## Introduction

Urology has long been recognized as an avid adopter of new technologies and innovations in surgical practice. In concert with the exponential and rapid improvements in laparoscopic techniques and instrumentations over the last two decades, urologists’ enthusiasm to implement minimally invasive approaches has led to the near extinction of open surgical approaches in several different urological diseases. This captivation was driven mainly by the morbidity associated with classic open approaches and the real benefits of less invasive approaches.

Since the description of the first laparoscopic nephrectomy by Clayman *et al.* ^1^ in 1991, there has been a continual effort to enhance outcomes and introduce newer, less invasive approaches. This has been accomplished through laparoendoscopic approaches which encompass a wide array of surgical interventions, including robotic surgery, laparoendoscopic single-site surgery (LESS), and natural orifice transluminal surgery (NOTES). The aim of this review is to highlight the major conceptual advancements in this field regardless of both the specific surgical approaches, whether pure laparoscopic or robotic, and the specific organ or pathology treated.

This review was conducted through a comprehensive literature search in order to describe the major advances that impacted daily practice or had the potential to do so. No specific search period was applied. All relevant articles that represented a key addition to existing knowledge were selected on the basis of the discretion of the authors. For descriptive purposes, these advances will be classified into the following categories: enhanced laparoscopic visualization, augmented reality in laparoscopy, overview of access, and new advances in insufflation devices.

## Enhanced visualization

One of the main advantages of laparoscopy is the enhanced appreciation of intraoperative anatomy through the use of magnifying optics with high-definition properties. Although this greatly facilitates certain parts of each procedure, this comes at the cost of losing some or all of the haptic feedback because of the presence of an instrument interface between the surgeon’s hands and the surgical field. Haptics generally describes touch feedback, which is a combination of kinesthetic (force applied to muscle and joints) and cutaneous (tactile; applied to sensory receptor on the skin) feedback ^1^. In laparoscopic surgery, the surgeon feels the interaction of the instrument and the tissue via the shaft of the instrument. Thus, the force feedback is partially maintained while there is no tactile feedback as he is not touching the tissue directly with his fingers as in open surgery. On the other hand, lack of haptic feedback is profound in robot-assisted surgery because of the lack of both force and tactile feedback. Recently, the TELELAP ALF-X robotic surgical platform (Sofar SpA, Milan, Italy) was introduced; this platform provides force feedback but no tactile feedback and was used in gynecological procedures such as ovarian cystectomies and hysterectomy ^2, 3^. To the best of our knowledge, this system was not used in urology other than in preclinical trials ^4, 5^. Of note, the newest generation of the commercially available robot, the da Vinci Xi (Intuitive Surgical, Inc., Sunnyvale, CA, USA), provides visual feedback relative to the degree of pressure applied between the jaws of some of the available instruments, such as the Vessel Sealer™ and the Endowrist stapler™ (Intuitive Surgical, Inc., Sunnyvale, CA, USA).

It is a well-established observation that minimally invasive surgeons gradually develop alternative visual cues in order to compensate for the lack of tactile feedback ^6^. However, the mere utilization of high-definition cameras does not help completely overcome this limitation. Recently, several three-dimensional (3D) cameras and monitors have been introduced and used in conventional laparoscopy and were shown to demonstrate a high degree of accuracy and precision in conducting the surgical procedure in a more efficient manner ^7– 10^. With regard to robotics, the 3D visualization was a feature that accompanied robotic surgical platforms since its inception and to the best of our knowledge there were no data to compare between the 2D and 3D visualization in robotics.

In robotic surgery, real-time ultrasound (US) visualization with simultaneous display at the console can be performed by using the TilePro technology (Intuitive Surgical, Inc.). This can be used with a variety of ultrasound probes that are used at the bedside by the assistant or the surgeon (through a robotic instrument). Several examples exist for the use of this technology and one of the earliest uses was when a transrectal US was used during radical prostatectomy to visualize the neurovascular bundle, shape of the prostate, and surgical instruments to help guide various surgical steps of the procedure in both laparoscopic and robotic prostatectomy ^11^. In one report that used this technology in robotic prostatectomy, the transrectal probe was manipulated with a remote controller without the need of a bedside assistant ^12^. Although the use of this system demonstrated less positive surgical margin rates in T3 prostate cancer, it did not gain widespread usage during robot-assisted radical prostatectomy since most surgeons are currently familiar with laparoscopic prostate anatomy and can use alternative visual cues to guide them during these surgical steps. On the other hand, the use of robotically held, small linear probes with 13.3 Hz is of clinical value, especially during robot-assisted partial nephrectomies. The images can still be displayed on the console and help identify endophytic tumors and plan accurate surgical resection. In our opinion, the use of intraoperative US in partial nephrectomy offers a great technical advantage in accurate tumor localization and subsequently may decrease operative time. However, its impact on surgical margins and oncological outcomes is not clear. Although certain reports demonstrated less positive surgical margins in partial nephrectomies when intraoperative US was used ^13^, this could be attributed to surgical skills and experience.

One of the limitations of intraoperative US is that the surgeon has to correlate the US imaging with the real images of the anatomy through cognitive fusion. For example, the depth of a tumor can be only mentally estimated, with the potential for error, resulting in cutting through a tumor or much deeper, leading to unnecessary loss of healthy parenchymal tissue. Interestingly, certain reports described the benefit of combined *in vivo* and *ex vivo* US examination of surgical margins and found that this correlated with final histopathological margins ^14^. Veeratterapillay *et al.* recently demonstrated an interesting concept of overlaying the intraoperative US images on the surgical field, resulting in direct fusion of the US and the 3D images seen in the console ^15^. However, the authors reported some delay resulting from processing of these high-definition images.

## Augmented reality and surgical navigation

Augmented reality (AR) has been an active area of research and development in laparoscopic urology in recent years. It is an advanced form of image-guided surgery and implies the use of enhanced visual information to the normal surgical field to supplement the lack of tactile feedback. This information is typically extrapolated from preoperative or, less commonly, intraoperative 3D imaging from US, computerized tomography, or magnetic resonance imaging ^16– 19^. The images are superimposed on the real-time surgical field to guide precise surgical resection. This was first reported by Marescaux *et al.* in a laparoscopic adrenalectomy in 2004 ^16^.

The most common operation in which AR is used in urology is partial nephrectomy, where understanding of hilar anatomy and 3D location of the tumor is of paramount importance. This is specifically used to avoid vascular injuries, collecting system violation, and incomplete tumor resection in partial or complete endophytic tumors. AR starts with “registration”, where multiple points from both the images and the real anatomic physical objects are aligned in a single coordinate system ^20^. There are various methods of registration and this can be done manually or with computer-assisted surface-based methodologies, fiducial based registration, stereotactic image registration, or a combination of these methods ^19, 21– 24^. The details of these techniques are beyond the scope of this review.

One of the main challenges of AR is organ movement and tissue deformation during respiration or surgical manipulation. The real-time changes in organ size and shape during surgery may significantly impair the accuracy of image fusion. So far, no real practical solution exists for this problem and this precludes the use of AR in clinical practice. However, in an attempt to do so, Teber *et al.* used an intraoperative mobile C-arm computed tomography scan and custom-designed navigation aids to enhance surgical decision making immediately prior to surgical resection of kidney tumors ^18^. The model was tested in 10 porcine renal units and 10 laparoscopic partial nephrectomies. They demonstrated a small margin of error of 0.5 mm, and all specimens had negative surgical margins. Although this is encouraging, the impact of this technique on the perioperative and oncological outcomes remains to be proven.

Surgical navigation in laparoscopy can be aided with the use of various injectable substances that can be visualized intraoperatively. The most commonly used material so far is indocyanine green (ICG) dye, which is a fluorescent dye that was first used in the 1950s at the Mayo Clinic for medical diagnostic purposes in hepatology and cardiology. It is a fluorescent substance that is administered intravenously and binds to plasma proteins (globulin and plasma protein); thus, it is confined to the intravascular compartment with a half-life of 4 minutes and complete hepatic excretion. ICG dye has no major adverse effects and has a peak spectral absorption of 800 nm ^25– 31^. It can be visualized with a special near-infrared camera (700–1000 nm) that is currently available in both laparoscopic and robotic platforms: the Storz D-light (Storz GmbH, Tuttlingen, Germany) and Firefly fluorescence, the latter of which is incorporated directly into the da Vinci Si and Xi system (Intuitive Surgical, Inc.). The vessels and vascular organs can be seen as bright florescent structures, and renal cortical tumors (except oncocytomas, which will appear as isofluorescent) will be less bright as they have reduced expression of ICG dye carrier protein. The main application of ICG dye in urology is in partial nephrectomy for selective clamping and localization of the tumor, and in order to decrease warm ischemia time. The ability of this technique to differentiate tumor from parenchyma is reported to range from 65 to 10% with a positive surgical margin rate of 0 to 6.4% and warm ischemia time of 12.5 to 26.6 minutes ^32^. Although the aim of this technique is to aid in selective clamping, the impact of selective clamping on long-term renal function and epidermal growth factor receptor (eGFR) reduction needs further evaluation.

Although this article focuses on what we see to be the main advances in laparoscopic urology, it is worth noting the major expected role of 3D printing in surgery in general and its impact on laparoscopy. 3D printing is part of a process called additive manufacturing in which an object is created by adding materials layer by layer. This involves scanning an existing object using sophisticated software (or occasionally creating the image of an object), and then converting these images into multiple successive 2D layers that can be deposited by the printer and added together to form a 3D object again ^33, 34^. Materials of different physical properties can be used in 3D printing to serve different purposes. One of the uses of this technology is preoperative planning for complex surgical operations. Silberstein *et al.* ^35^ and Zhang *et al.* ^36^ reported the use of 3D printed kidney units with cancer to educate patients and trainees prior to partial nephrectomies. Interestingly, the authors used the cross-sectional images to build these models.

An additional use of 3D printing is to reproduce surgical instruments where several studies reported the use of 3D-printed surgical instruments in animal and cadaveric models such as laparoscopic ports or ureteral stents ^37, 38^. Among the various advantages of these instruments, the benefits of cost and potential individualization are very promising.

Lastly, the ultimate goal of 3D printing is to use biological material to produce biological grafts or transplant artificial organs (bioprinting). This will revolutionize the treatment of many urological disorders. However, this is currently facing many challenges that are beyond the scope of this review.

## Insufflation devices

The creation of a large and stable working space between the abdominal wall and viscera is essential for successful conduction of any laparoscopic procedure. The most commonly used method is the insufflation of carbon dioxide (CO _2_) to establish pneumoperitoneum. CO _2_ is used to achieve the intra-abdominal pressure and space that can be tolerated by the patient without adverse physiological effects. CO _2_ is commonly used because it is inexpensive, colorless, odorless, nonflammable, and rapidly eliminated from the systemic circulation ^39^. Effective and stable pneumoperitoneum can result in shorter and safer operation. With conventional insufflators, laparoscopic ports are supplied with mechanical valves that help maintain pressure and prevent leakage while the insufflator is set to automatically maintain pressure at a certain level. A relatively recent innovative insufflator was introduced as the AirSeal system (Surgi-Quest, Inc., Milford, CT, USA). This system has the potential advantages of smoke evacuation, gas circulation, and maintenance of a more stable high-flow pressure that rapidly replaces the sudden decrements in pressure in case of leak. The system achieves these aims through a valveless trocar and tri-lumen filter tube set. The gas leakage is prevented through tiny circumferential CO _2_ nozzles within the proximal part of the trocar that creates an invisible gas “curtain” that has higher pressures than the abdominal wall. The tubing is composed of three lumens for inflow, outflow, and constant pressure monitoring; all are connected to a built-in filter that helps eliminate smoke and microbial contamination and potentially decrease CO _2_ consumption. The circulation of gas takes place at a rate of 3 L/min and reactively increases in case of pressure drop.

The use of this insufflation system was found to be associated with shorter operative times and lower CO _2_ consumption and, more importantly, reduced patient systemic CO _2_ absorption. Horstmann *et al.* prospectively compared the conventional insufflation system with the AirSeal and found that the latter resulted in more stable pneumoperitoneal pressures and easier specimen and needle extraction because of valveless ports ^40^. In our experience, we found that the majority of pelvic urological procedures can be performed safely in the hands of experienced surgeons without the added cost of this device to the operating room. Moreover, the benefit of this insufflation system is evident mainly in cases of partial nephrectomy where continuous suction is needed during renorrhaphy. Of note, this insufflation system mandates the use of large-size trocars and results in noise from the high flow of insufflation that may make direct verbal communication between the assistant and the console surgeon somewhat difficult in robotic cases.

## Access

The evolution of surgical intervention from classic open surgery to minimally invasive laparoscopic surgery resulted in the development of less invasive techniques. This was manifest in the description of LESS, minilaparoscopy, and NOTES. In LESS, surgery is performed through a single port of entry to the abdominal cavity in an attempt to minimize pain, enhance recovery, and improve cosmesis. In contrast, natural orifice surgery (NOTES) is where surgery is performed in a scar-less fashion through transoral, transvaginal, transrectal, or transurethral routes. Although these techniques of access represent an attractive option to perform some of the most commonly performed procedures in urology, its widespread adoption is limited by the technical difficulty encountered and the need for the development of more sophisticated flexible instruments that can facilitate these surgical tasks. Moreover, the measurable improvement of patient outcomes needs to be objectively validated to demonstrate superiority over standard laparoscopic and robotic procedures.

Since the first NOTES nephrectomy in a porcine model by Gettman *et al.* was described in 2002, there have been several other reports of pure and hybrid NOTES urological procedures in the literature ^41^. Most of these efforts were preclinical and were performed on either animals or cadavers and focused on NOTES nephrectomy with the less commonly described NOTES prostatectomy ^42– 59^. The first clinical NOTES procedure was performed by Branco *et al.*, who successfully performed a hybrid transvaginal nephrectomy ^60^. Kaouk *et al.* performed the first pure NOTES nephrectomy in 2010 through a 3 cm colpotomy incision followed by the first robot-assisted hybrid NOTES donor nephrectomy in 2012 ^61, 62^.

Although a transurethral prostate resection for benign disease is the current standard of care, performing a radical prostatectomy through a transurethral approach is challenging. This was first reported by Humphreys *et al.* in both a cadaveric and a clinical setting ^63, 64^. The latter was performed in two patients without perioperative complications and with negative surgical margins. Further development of the procedure was hindered by the lack of an appropriate transurethral anastomotic device.

Despite the great promise of pure NOTES, the surgical instrumentation has been criticized because of lack of flexibility, difficult retraction of large and heavy intra-abdominal organs, and difficult use of relatively large hemostatic devices. These limitations may make surgery extremely difficult, especially when a complication such as bleeding occurs. Additionally, closure of site of entry is of extreme importance, especially in transgastric, transvesical, and transcolonic approaches where poor closure carries a great risk of infectious complication.

Each natural orifice route is associated with its own advantages and disadvantages. The transvaginal route is most commonly used because of the advantage of easy closure and low contamination risk but is associated with gender confinement to 50%. Digestive tract access is associated with easier access to kidney (in transgastric access) and pelvis (in transcolonic and transrectal access) but carries a significant risk of contamination and wound closure and specimen extraction difficulty.

Owing to the aforementioned difficulties associated with pure NOTES, many groups use a hybrid NOTES technique in which a number of laparoscopic instruments (usually one or two 5 mm ports) are added to the instruments inserted through the natural orifice. The prevailing thought was that by avoiding the extraction incision the morbidity would be reduced. Thus, using the natural orifice for specimen extraction (most commonly, the vagina) provides a potential cosmetic and recovery advantage ^65^.

## Conclusions

Laparoscopic and robot-assisted approaches in urology have fostered significant advances in minimally invasive surgery and in some instances completely replaced previously performed standard open procedures such as robotic prostatectomy and laparoscopic live-donor nephrectomies. Although efforts continue to explore newer, less invasive technologies and procedures, their widespread implementation will depend on the introduction of newer instrumentations that facilitate these surgeries. In order to prove the clinical utility of these newly described technologies and their equivalent therapeutic benefits compared with conventional laparoscopy, there is a strong need to have an objective and stringent evaluation of its clinical outcome.
